# Effect of the Kinesio tape to muscle activity and vertical jump performance in healthy inactive people

**DOI:** 10.1186/1475-925X-10-70

**Published:** 2011-08-11

**Authors:** Chen-Yu Huang, Tsung-Hsun Hsieh, Szu-Ching Lu, Fong-Chin Su

**Affiliations:** 1 Department of Biomedical Engineering, National Cheng Kung University, 1 University Road, Tainan, (701), TAIWAN; 2 Medical Device Innovation Center, National Cheng Kung University, 1 University Road, Tainan, (701), TAIWAN

## Abstract

**Background:**

Elastic taping applied on the triceps surae has been commonly used to improve the performance of lower extremities. However, little objective evidence has been documented. The purpose of this study was to investigate the effect of elastic taping on the triceps surae during a maximal vertical jump. It was hypothesized that elastic taping to the triceps surae would increase muscle activity and cause positive effect to jump height.

**Methods:**

Thirty-one healthy adults (19 males and 12 females with mean age, body weight and height for 25.3 ± 3.8 years old, 64.1 ± 6.2 kg, and 169.4 ± 7.3 cm, respectively) were recruited. All participants performed vertical jump tests prior to (without taping) and during elastic taping. Two elastic tapes, Kinesio tape and Mplacebo tape from two different manufacturers, were applied to the participants, respectively.

**Results:**

The results showed that the vertical ground reaction force increased when Kinesio tape was applied even when the height of jump remained about constant. However, the height of the jump decreased, and there was no difference on the vertical ground reaction force in Mplacebo taping group. Although the EMG activity of medial gastrocnemius tended to increase in Kinesio taping group, we did not see differences in EMG activity for the medial gastrocnemius, tibialis anterior and soleus muscles in either group.

**Conclusions:**

Based on the varied effects of Kinesio tape and Mplacebo tape, different intervention technique was suggested for specific purpose during vertical jump movement. Mplacebo tape was demanded for the benefits of stabilization, protection, and the restriction of motion at the ankle joint. On the other hand, the findings may implicate benefits for medial gastrocnemius muscle strength and push-off force when using Kinesio tape.

## Background

Vertical jumping is a kind of movement often seen in sports and exercise skill tests, and it has been discussed frequently in past related studies [1-4]. In most situations, before the push off movement begins, vertical jumping is carried out by the rapid extension of the hip, knee, and ankle joints [2]. Vertical jumping height is often demanded in the performance of sports and is an ability usually used in the test for basic capability to engage in sports or exercise [5].

Motion analysis provides a detailed picture of the muscular effort expended at the joints during the performance of exercise [6]. Several studies have attempted to identify mechanisms to which performance of the vertical jump can be attributed and have sought to improve the performance of athletes. Some authors have reported that the increased output of maximal force during the concentric phase is due to the increased neural drive from the muscle [7,8]. It has been suggested that vertical jump height performance could be improved because of the increased muscle contraction force [9].

In order to improve performance in sports, several intervention techniques are applied to athletes. For example, a cutaneous stimulation to enhance muscle contraction has been widely used in rehabilitation and in sports. However, the effect is not long-lasting, with most overflows continuing only about 15-30 minutes after cessation of treatment. So far, no modalities or externally applied dressings have been proven to offer more prolonged treatment effects. An elastic tape applied at the same time might cause proprioceptive stimulation such that the enhancement of improved joint range of motion and thigh muscle function during exercise might not be limited [10]. Many researchers have pointed out effects that include postural amendment, feelings of comfort, decreased inflammation and pain, and the normalization of joint range of motion when elastic taping was involved [11,12].

However, sticking to the underlying mechanism, the functions of elastic tape have remained in question and there are few papers focusing on the beneficial results of elastic tape on a specific movement associated with vertical jumping. An increase in the stability of the body or extremities, support or protection of the joint, the correction of the alignment of the body or limbs, the modification of the biomechanics of movements and the promotion of sensor-motor functions like the proprioception influence and insignificance sensory input inhibition might be reasons that have contributed to the effects of elastic taping [13-15].

Adhesive tapes are also frequently considered for use on athletes. Both types, non-elastic tape and elastic tape, can be considered as a whole. Non-elastic tapes are usually chosen to rectify misalignments of the body or extremities, or they serve a support function. Non-elastic tape is referred to as Athletic tape, Leukotape, or Mplacebo tape. Mplacebo tape provides comfortable sensory on skin but it is not extensible so that it may cause a shear force damage to skin. Adhesive tape has been most commonly used on the athletic field and in clinical therapy, respectively. However, depending on the specific materials non-elastic tapes are made of, they may provide a strong adhesion force leading to a restriction of a body segment's movement or cause skin discomfort. On the other hand, the principle function of elastic tape is to give an effective force through minimal contact with the skin and to normalize the motion of the body or extremities.

Elastic tape is usually applied on an athletic field or during rehabilitation therapy. Kinesio tex is a kind of elastic tape devised by Dr. Kase (1980) that has been popularly in use in Asia for quite some time [16]. Kinesio tex is very different from other traditional elastic tapes because it is manufactured with a special weave and viscosity that allows ventilation and water resistance, with more expanded elasticity and a minimization of skin discomfort. The taping methods using Kinesio tex developed by Kase have been suggested for the beneficial effects of the tapes and possible useful mechanisms that include physical corrections, fascia relaxation, space recuperation, ligament and tendon support, movement rectification and lymphatic fluid circulation [16]. Even though these hypotheses have not been proven so far, Kinesio taping is being used more and more in clinical, rehabilitation and orthopedic departments as well as on athletes. The elimination of perspiration, freedom of motion and a smooth feeling are particular properties of Kinesio taping that have been shown to be desirable to athletes. One report by Murray in 2001 pointed out that using Kinesio taping on the quadriceps muscles caused an improvement in joint range of motion in the knee, animated EMG activities and increased the quadriceps femoris's muscle strength for two patients [10]. They had anterior cruciate ligament reconstruction before the test. However, these points have never been examined by any exact and carefully designed study.

Authors also have focused on the study of the functions of non-elastic tapes. These investigations have shown clearly that there were no significant differences with regard to the benefits of non-elastic tapes acting on muscle activities around the scapula as detected by electromyographic signs [13,14]. Furthermore, the Hoffmann reflex could be stimulated as gastrocnemius muscle contraction elevated by dorsiflexion of the ankle or stretching the Achilles' tendon, and Hoffmann reflex of the muscle may be restrained by this kind of adhesive tape [17]. The only benefits indicated have been associated with the reduction of pain and an increase in the range of motion [11,18].

Research concerning elastic taping attached on triceps surae muscle during vertical jumping is rare in previous literature, even though elastic tape applied on the triceps surae has been commonly used by athletes to improve the performance of lower extremities. We hypothesized that elastic taping to the triceps surae would increase such muscle activity and cause positive effect to jump height. Therefore, the purpose of this study was to investigate the effect of elastic taping on the triceps surae during a maximal vertical jump by examining the vertical ground reaction force (VGRF), jump height, and electromyographic (EMG) activity of the medial gastrocnemius (MG), tibialis anterior (TA), and soleus (Sol) muscles.

## Methods

### Participants

Thirty-one healthy adults (19 males and 12 females) age from 21 to 31 years old were recruited in this study. They were completely inactive without habit of regular exercise before the study. Before formal testing, the evaluations of based on the clinical criteria in lower extremities were assessed by well trained physical therapist. Subjects were excluded if they had a history of spinal, hip, knee or foot pathology, any neurological impairment or a history of lower limb fractures. Their average age, body weight and height were 25.3 ± 3.8 years old, 64.1 ± 6.2 kg, and 169.4 ± 7.3 cm, respectively.

### Experimental design

Before the data collection, all participants were informed about the study processes and then were asked to sign a consent form approved by the Institutional Review Board of National Cheng Kung University Hospital. All participants executed the vertical jump tests prior to and during the elastic taping. Two tapes, elastic tape A (Kinesio Tex KT-X-050, Tokyo, Japan) and non-elastic tape B (Micropore, 3 M, St. Paul, USA), were applied to the participants, respectively.

**Figure 1:**
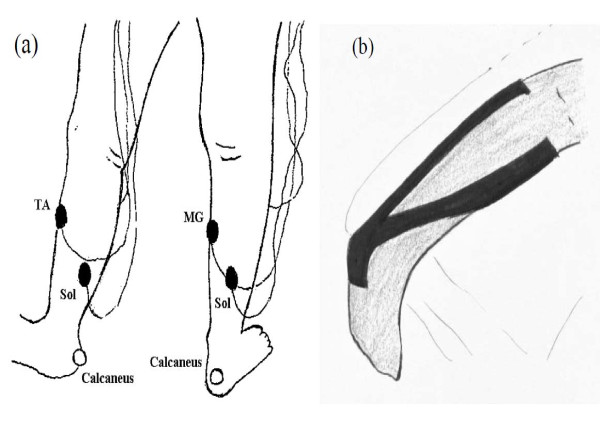
**The attached positions of EMG electrode and reflector**. (a) Electromyographic (EMG) activity was recorded from the medial gastrocnemius (MG), tibialis anterior (TA), and soleus (Sol) muscles using Ag-AgCl electrodes. Jump height was collected by kinematics data of reflector attached on calcaneus bone. (b) The elastic taping technique used in this study.

For the elastic taping technique, we used a Y shaped Kinesio tape (Kinesio Tex KT-X-050, Tokyo, Japan) for calf muscle taping according to the recommendation of Kase (1980). First, the proximal head of Y shaped Kinesio tape was applied on the surface of calcareous bone on the sole of the foot with the subject in a relaxed prone position. Then, two distal heads of Y shaped Kinesio tape were attached following the soleus muscle and ended on the surfaces of medial and lateral gastrocnemius muscles below the knee joint, respectively (Figure 1-b). The same sized Y shaped Mplacebo Micropore tape was applied over the calf muscle.

Before doing sports activities, warm up procedures are often recommended to promote performance and prevent injury. Easy, gentle movement has been suggested for jumping rather than proprioceptive neuromuscular facilitation or static stretching exercise, both of which could decrease muscle force production [19,20]. After placement of the electrodes, the subjects walked on a treadmill at 1.34 m/s, the mean fast walking speed of total subjects, for 5 minutes as the warm up period. Before performing the vertical jump, the subjects were instructed in the proper jumping technique. This involved performing a 2-legged upward vertical jump with both feet on the force plate with the subject's maximal effort with the bilateral hands placed on the hips [21]. To reduce training effects, subjects were initially allowed to practice until they were full adapted and familiar with the protocol.

After the brief warm-up and practice, participants performed the vertical jump test in the following sequence: first, the subject performed five trials of a maximal vertical jump as the baseline trials; second, the elastic tapes were applied over the bilateral triceps surae muscles by an experienced physical therapist while subjects were blinded for taping type; third, to let participants obtain full resting and avoid muscle fatigue induced by the previous trials, after a period of 30 minutes with the tape, the another five trials were tested.

Baseline vertical jump performance was assessed on every test session. Upon arriving for a test session, subjects were fitted with EMG surface electrodes. A pretest/posttest repeated measure design was used to understand the effects of Kinesio and Mplacebo taping during the performance of the maximal vertical jump. Every subject applied both A and B tapes. The test sequence of tape A and tape B was randomly processed for each subject to avoid bios. Two taping sessions were performed at an interval of at least 3 days to avoid accumulation of the taping effects.

### Data recording

The jump height was measured using a video-based motion analysis system (Motion Analysis Corporation, Santa Rosa CA, USA). Kinematic data was collected using an eight-camera motion analysis system with the sampling rate set at 100 Hz. Reflective marker was placed on the dorsal aspect of the calcaneus bone on the bilateral legs to collect the jump height.

The vertical ground reaction force (VGRF) was measured using a force platform (9281B, Kistler Instrument Corporation, Amherst, NY, USA) at a sampling rate of 1000 Hz. The electromyographic (EMG) activity reflects the signal of active muscle fibers [22]. It was used to record from the medial gastrocnemius (MG), tibialis anterior (TA), and soleus (Sol) muscles using Ag-AgCl electrodes (MA-300 EMG system, Motion Control, USA) at a sampling rate of 1000 Hz during the whole jump task (Figure 1).

### Data analysis

In kinematics, the coordinate data were then smoothed with a 2nd order forward and backward Butterworth filter with a cut off frequency of 6 Hz, and the displacement was calculated. The cut off frequency was chosen from the results of the residual analysis performed on the data [23].

**Figure 2:**
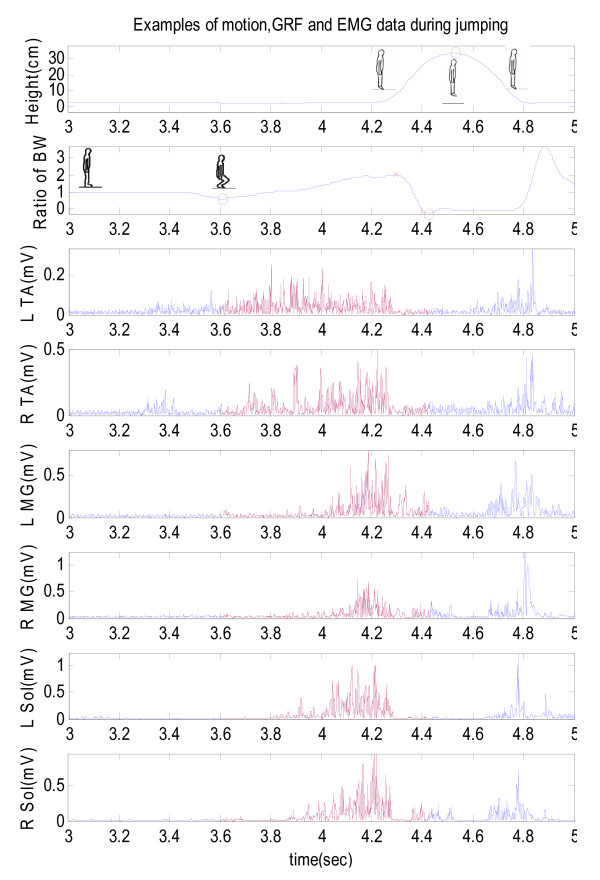
**Example of result in a trial**. Example of result in a trial. The 1^st ^box, the motion (height) during vertical jump; 2^nd ^box, VGRF during vertical jump. The VGRF were normalized by subject' own weight during vertical jump; 3^rd ^to 8^th ^box, EMG data during jumping.

The EMG signals were band-pass filtered from 8 to 1000 Hz [4]. The EMG value was collected with the duration from minimal to maximal GRF during lower extremity concentric contraction to push off (Figure 2, red trajectory), then calculated using a moving average window of 31 ms [4]. VGRF data from the force platform were digitally filtered using a bi-directional, low-pass, fourth-order, Butterworth filter, with a cut-off frequency of 7 Hz. Displacement data from the cable-extension transducer were filtered similarly with a cut-off frequency of 7 Hz [4]. Finally, the individual VGRF was normalized by the body weight, as a new parameter (ratio of body weight) to analyze the tapping effect on performance of VGRF (Figure 2, second layer).

Statistical analysis was undertaken using SPSS version 10.0 (SPSS Inc., USA) with significance level defined as *p *< 0.05 for each test. All data were presented as average ± standard error of the mean (SEM). For the changes in the vertical jump height, vertical ground reaction force and EMG at before and after taping, a repeated measures analyses of variance (ANOVA) was used with time as within-subjects main factor. Pair-t test was used as post-test to compare before and after taping when the main effect of was significant. Also, the effects of dependent variables in different types of taping, the independent t-test was performed to determine the contributions of individual taping effects.

## Results

Figure 2 shows a trial example during vertical jump (figure. 2). The vertical ground reaction force had been normalized by each subject's body weight for the ratio of the VGRF. These values in group A were 2.28 ± 0.07, 2.31 ± 0.08 before and after applying the Kinesio tape, respectively. In group B, they were 2.31 ± 0.07 and 2.32 ± 0.07 on the baseline and during the period of use of the Mplacebo tape. There was a significant time main factor (F(1,14) = 6.23; p = 0.03*) in group A, but not in time factor in group B (F(1,15) = 0.10; p = 0.75).

The jump height went to 39.67 ± 1.70 cm and 40.29 ± 2.04 cm (p = 0.86) before and after the Kinesio tape was used, respectively (group A). The results were 39.44 ± 2.28 cm and 38.01 ± 2.37 cm (p = 0.03*) in the same event while the Mplacebo tape was considered. Repeated measure of ANOVA analysis for time effect demonstrated not reach to significant in group A (F(1,14) = 0.44; p = 0.52), but showed significant in group B (F(1,15) = 6.51; p = 0.02). The post pair-t test showed that the ratio of the VGRF significantly increased (p = 0.02*) in group A but not change in group B (p = 0.86). However, the height of jump remained approximately the same (p = 0.86) in group A. In contrast, the height of the jump showed a significant decrease (p = 0.03*). In the control group without taping, no significant differences were found in all parameters across the time when compared to pre baseline data (all p > 0.05) (table 1). Furthermore, to compare with different types of taping after taping, the independent t-test failed to reveal any significant either for VGRF (t = -0.68, p = 0.95) or jump height (t = 0.72, p = 0.48).

**Table 1:** Comparison of vertical jump height and ratio of VGRF pre and post taping in groups A & B

	Control group			Group A			Group B		
	**Pre** **(mean ± SE)**	**Post** **(mean ± SE)**	**p-value**	**Pre** **(mean ± SE)**	**Post** **(mean ± SE)**	**p-value**	**Pre** **(mean ± SE)**	**Post** **(mean ± SE)**	**p-value**

Ratio of VGRF	2.30 ± 0.30	2.29 ± 0.06	0.72	2.28 ± 0.07	2.31 ± 0.08	0.02*	2.31 ± 0.07	2.32 ± 0.07	0.64
Jump height (cm)	39.56 ± 2.23	39.60 ± 2.04	0.91	39.67 ± 1.70	40.29 ± 2.04	0.86	39.44 ± 2.28	38.01 ± 2.37	0.03*

*p < 0.05

The values of the EMG activity were normalized by the base values pre-applying the two types of tape on each subject. Thus the pre-application and post-application ratios of EMG activity were the determinants for comparison. The outcome of the pre-post ratios of the left tibialis anterior, right tibialis anterior, left medial gastrocnemius, right medial gastrocnemius, left soleus and right soleus muscles EMG activity were 1/0.97 ± 0.09 (p = 0.27), 1/1.00 ± 0.19 (p = 0.97), 1/1.08 ± 0.11 (p = 0.02*), 1/1.07 ± 0.10 (p = 0.02*), 1/1.02 ± 0.09 (p = 0.36), and 1/1.01 ± 0.08 (p = 0.59) in group A, respectively (table 2). In group B, they were 1/1.01 ± 0.15 (p = 0.67), 1/0.95 ± 0.25 (p = 0.67), 1/1.00 ± 0.09 (p = 0.30), 1/1.04 ± 0.09 (p = 0.10), 1/1.04 ± 0.10 (p = 0.43) and 1/0.98 ± 0.10 (p = 0.37). The EMG activity of the medial gastrocnemius muscle significantly increased in group A, but we could not see differences in the EMG activity for the other muscles in group B (table 2).

**Table 2:** Pre and post taping comparison of EMG activity in groups A & B

	Control group			Group A			Group B		
	**Pre-Post ratio(ratio ± SE)**		**p-value**	**Pre-Post ratio(ratio ± SE)**		**p-value**	**Pre-Post ratio(ratio ± SE)**		**p-value**

L. TA	1	0.99 ± 0.06	0.77	1	0.97 ± 0.09	0.27	1	1.01 ± 0.15	0.67
R. TA	1	0.96 ± 0.11	0.86	1	1.00 ± 0.19	0.97	1	0.95 ± 0.25	0.67
L. MG	1	1.04 ± 0.07	0.48	1	1.08 ± 0.11	0.02*	1	1.00 ± 0.09	0.30
R. MG	1	1.01 ± 0.12	0.07	1	1.07 ± 0.10	0.02*	1	1.04 ± 0.09	0.10
L. Sol	1	0.97 ± 0.07	0.39	1	1.02 ± 0.09	0.36	1	1.04 ± 0.10	0.43
R. Sol	1	0.98 ± 0.08	0.42	1	1.01 ± 0.08	0.59	1	0.98 ± 0.10	0.37

*p < .05

## Discussion

Based on this study, differences had been observed when applying Kinesio and Mplacebo tape. The results showed that vertical ground reaction force (VGRF) and EMG activity of medial gastrocnemius significantly increased during the jumping task when Kinesio tape was applied. The statistical analyses showed that there were no significant differences in jump height in the Kinesio taping group. However, the Mplacebo taping caused a significant decrease in jump height and no change in EMG activity of all testing muscles and VGRF. Different types of elastic tape caused varied effects during exercise activity. The distinct structure and degree of elasticity would produce different biomechanical effects.

Tape with lower degrees of elasticity, such as Mplacebo tape, cause similar effects to rigid ones. Specifically, it supports or stabilizes the selected muscle and restricts the motion of the joint [24]. Thus, ankle dorsiflexion would be limited during the motion in which lower extremity squatted down to prepare pushing off. In addition, instead of the expected facilitating effect, Hsu et al. (2009) also addressed the decrease in activity of the lower trapezius muscle with Mplacebo taping [25].

Opinions about the effects of Kinesio taping on muscle activity are inconsistent among previous studies. There is report making conjectures that kinesio tape caused no influence on muscle activity for young athletes [26]. Even for the non athletes, the results of this study reversed the negative suggestion made by Herrington (2004) of significantly decreased muscle activity [27].

On the contrary, Kinesio taping had been shown to facilitate muscle effort on the muscles to which the tape is applied. The effects of Kinesio tape in our study were similar to the reports by a number of other researchers. For instance, Hsu et al. (2009) noted positive effects on both muscle activity and motion performance of scapular after Kinesio taping [25]. Furthermore, Kinesio tape was suggested to cause an improvement in certain modalities in clinical applications. Another report mentioned the specific effect as the modulation of the skin mechanoreceptor. Additionally, after using Kinesio taping on two anterior cruciate ligament reconstruction patients, the knee joint extension angle increased [10]. Since Kinesio tape has an elastic property, it permits free joint motion. Such tape could offer a means to increase joint loading and activity of the taped muscle, as well as to even out the movement and power of the joint during the performance of a vertical jump [28-30].

Regarding jump height, several factors are projecting to influence performance. For example, we chose to investigate the effect of elastic taping on the gastrocnemius muscles on the basis of its essential and power role for ankle plantar flexing motion during jumping [31]. Based on our observation, it was suggested that Kinesio taping seemed to increase the muscle activation of the medial gastrocnemius muscle. With the exception of the medial gastrocnemius muscle, there were no positive effects with the application of tape to the tibialis anterior and soleus muscles in the Kinesio group.

Tibialis anterior and soleus muscles act to dorsal flex and plantar flex the ankle joint, respectively. The tibialis anterior should not provide the main effort to jump. The soleus muscle only passes the ankle, and the gastrocnemius crosses two joints, knee and ankle joints. When subjects prepared to push off, the knee extends to lock the origin of gastrocnemius. Thus, the gastrocnemius concentric contracted and could provide the maximal amount of strength, rather than the soleous muscle. Among a given muscle group, the muscle that contributes the most during an activity may reflect larger electromyography change with Kinesio taping. In an anatomically similar way, during the push off movement, medial gastrocnemius seemed to be the main muscle among triceps surae at ankle joint (figure. 2). Unfortunately it did not raise the jumping height.

Otherwise, although there was no statistically significant increase on the jumping height in post-test, the values still clearly showed a trend of minor improvement on the jumping height at the post-test when Kinesio tape was applied (40.29 ± 2.04 > 39.67 ± 1.70). As we know in the real sport world for example in high jump competition, every millimetre counts. Therefore a small improvement on jumping height after applying the Kinesio tape would be important for an athlete especially for a high jump athlete to enhance their performance record.

Synthesizing the phenomenon of muscle activity change and joint effects may also cause the results to raise the vertical ground reaction force. The phenomenon of increasing muscle activity was evident in our study that an improved VGRF occurred with Kinesio taping. Increases in VGRF and medial gastrocnemius activity could offer a means to increased Hoffman reflex amplitude [32] and motoneuronal excitability [33]. However, the results showed that jump height was not changed with the VGRF improvement after the use of Kinesio tape. The motoneuronal excitability may facilitate muscle strength, but not certainly or enough to increase exercise performance. The same result was shown in Firth's (2010) study. Although the hop distance was not changed, the triceps surae muscle was facilitated with Kinesio tape applied to the Achilles tendon [32].

Another factor to consider is that the duration of muscle taping may not be enough to produce greater muscle strength. Slupik et al. (2007) pointed out that the participation of a muscle's motor units increased maximally after twenty-four hours of Kinesio taping [33].

Additionally, the coordination and timing of segmental movements may also have played an essential role in achieving maximal vertical jump performance. The transfer of mechanical energy from the proximal to the distal segments of the leg in the vertical jump involves many muscle groups and joints. Therefore, the unchanged jump height in the Kinesio tape group may be due to the hip and knee extensors achieving a greater muscle effort at the maximal jump height than the ankle [6]. Further investigation should be designed to incorporate these muscle groups.

## Conclusions

In conclusion, there is not much information discussing the benefits with regard to muscle activity when applying elastic taping during a vertical jump. Our findings indicated an increase in muscle activity of the medial gastrocnemius during a maximal vertical jump immediately following Kinesio taping. Although the VGRF was increased after Kinesio taping, the jump height was not statistically improved. But value of jump height showed a trend of minor improvement after applying Kinesio tape. Compared to applications involving Kinesio tape, all muscle activities were not changed during jumping after the Mplacbo taping. The different patterns of muscle activity after taping may be due to different specialized weaves and viscosity.

A significant increase in the VGRF after the application of Kinesio elastic tape reflected that Kinesio taping might facilitate the muscle contraction capacity of the triceps surae during a vertical jump. Therefore, if muscle function excitement is considered for exercise or rehabilitation, Kinesio tape would be suggested rather than the Mplacebo tape used in this study. Performance tests on different subjects in future studies could provide more evidence in this area of interest.

### Applications and limitations

VGRF and EMG activity increased, but not jump height when the Kinesio tape was applied on triceps surae during the jumping task. In contrast, the Mplacebo taping did not affect the VGRF and EMG activity, but decreased the jump height.

Different effects were found in a maximal vertical jump exercise when various elastic tapes were used. Mplacebo tape is demanded for the benefits of stabilization, protection, and the restriction of motion at the ankle joint. On the other hand, the findings may implicate benefits for medial gastrocnemius muscle strength and push-off force when using Kinesio tape. Such intervention technique is suggested to enhance muscle contraction in rehabilitation and sports. The results of this study can not be generally applied to the population at large due to the limitations of age and health criteria of our subjects, as well as the selected exercise.

## Competing interests

The authors declare that they have no competing interests.

## Authors' contributions

All authors designed and performed the investigation. THH analyzed the data and CYH discussed the results. CYH and THH drafted and revised the manuscript and all authors read and approved the final manuscript.
